# Supplementary material for: A functional overlap between actively transcribed genes and chromatin insulator elements
Source: EMBO J. 2026 Mar 16;45(8):2587–613. doi: 10.1038/s44318-026-00730-2 (PMC13083895; doi:10.1038/s44318-026-00730-2)
Supplement: Supplementary file 1 — Appendix [file 44318_2026_730_MOESM1_ESM.pdf]

Appendix for A functional overlap between actively transcribed genes and chromatin insulator elements

Table of Contents

Appendix Figure S1: Mutated sequences of CTCF binding sites at the α globin locus 2

Appendix Figure S2: Editing strategy for introducing α-globin sequences into the ectopic site in the α-globin locus, to report effect on Hba-a1 expression 3

Appendix Figure S3: Extended characterisation of in vitro derived CD71+ cells with inserts. 4

Appendix Supplementary information: Limitations on interpretation of Capture-C from insertion models 5

Appendix Table S1: DNA sequences for sgRNAs, ssODNs and screening primers used for targeting CTCF binding sites at the mouse a-globin locus 6

Appendix Table S2: DNA sequences for sgRNAs and regions used for targeting the insertion site. 7

Appendix Figure S1: Mutated sequences of CTCF binding sites at the α globin locus

A

```
WT HS+44     1  ATTAGAAAGCCAGTGGCGCCACCTTGGGGCCCACTCTCAGCTGTTTATGACTACCGGGCA
Δ44-48       1  ATTAGAAAGCCAGTGGCGCCA---------------------------------------
WT HS+44    61  GGAGGTCTCTGGGGCGCCCCCTGCAGGCCACTATAAGTAGGTGCAAGGCTTTTCCAGTAT
Δ44-48      61  --------------------------------------AGGTGCAAGGCTTTTCCAGTAT

WT HS+48     1  CCGGAGATATAAGAAGGCGACGAGCACCCCCGTGTGGCGGGACTCGGAACTTCGCAGCTC
Δ44-48       1  CCGGAGATATAAGAAGGCGACGAGCACCCCCGTG------GACTCGGAACTTCGCAGCTC
```

B

```
WT θ1        1  CCTAGGCATCCTGGAACGATGCAGCGCCCCCTGGCGGCCTCTTGTGTTTCAGGACGTCTT
Δθ1          1  CCTAGGCATCCTGGAACGATGCAGCGGGATCC----GCCTCTTGTGTTTCAGGACGTCTT

WT θ1        1  CCTAGGCATCCTGGAACGATGCAGCGCCCCCTGGCGGCCTCTTGTGTTTCAGGACGTCTT
Δθ1θ2        1  CCTAGGCATCCTGGAACGATGCAGCGC-----------CTCTTGTGTTTCAGGACGTCTT

WT θ2        1  CCTAGGCATCCTGGAACGATGCAGCGCCCCCTGGCGGCCTCTTGTGTTTCAGGACATCTT
Δθ2/Δθ1θ2    1  CCTAGGCATCCTGGAACGATGCAGCGGGATCC----GCCTCTTGTGTTTCAGGACATCTT
```

A: Alignments show WT sequences of HS+44 and HS+48 CTCF binding sites, with the 20 bp core binding motif highlighted in blue (reverse orientation), and deleted sequences of HS+44 and HS+48 CTCF binding sites in Δ44-48 mice (77 bp and 6 bp deletions respectively).

B: Alignments show WT sequences of θ1 and θ2 CTCF binding sites, with the 20 bp core binding motif highlighted in blue (reverse orientation), and mutated sequences of θ1 CTCF binding site in Δθ1 (HDR mutation) and Δθ1θ2 mice (11 bp deletion), and the mutated sequence of θ2 CTCF binding in Δθ2 and Δθ1θ2 mice (HDR mutation in both models).

Appendix Figure S2: Editing strategy for introducing α-globin sequences into the ectopic site in the α-globin locus, to report effect on Hba-a1 expression

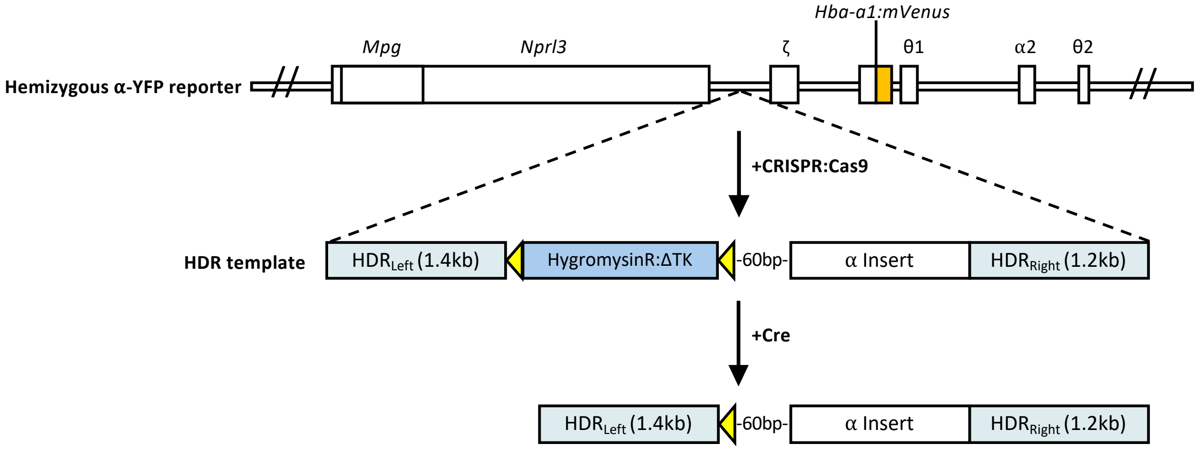

The insertion site is located between the enhancers and *Hba-x* gene (chr11: 32171787 - 32171856 mm9); ~2.6 kb from the R4 enhancer element. The *Hba-a1::mVenus* hemizygous (α-YFP reporter) line was generated previously (Tsang et al. 2024; Francis et al. 2022). *Hba-a1* was edited C-Terminus of the resulting α-globin protein is fused with 2A-peptide:mVenus:3xSV40-NLS. On the other allele (not shown) an 86kb deletion spanning the entire α-globin locus was introduced, such that it can be considered hemizygous for the α-globin locus. The insert sequences were introduced to the locus using CRISPR-Cas9 mediated HDR; the HDR template contains two arms homologous for either side of the insert site and a HygromycinR:ΔTK selection cassette flanked by homotypic loxP sites (yellow triangles), allowing cassette removal upon Cre-mediated recombination. For sgRNA and inserted sequences see **Appendix Table 2**.

Appendix Figure S3: Extended characterisation of in vitro derived CD71+ cells with inserts.

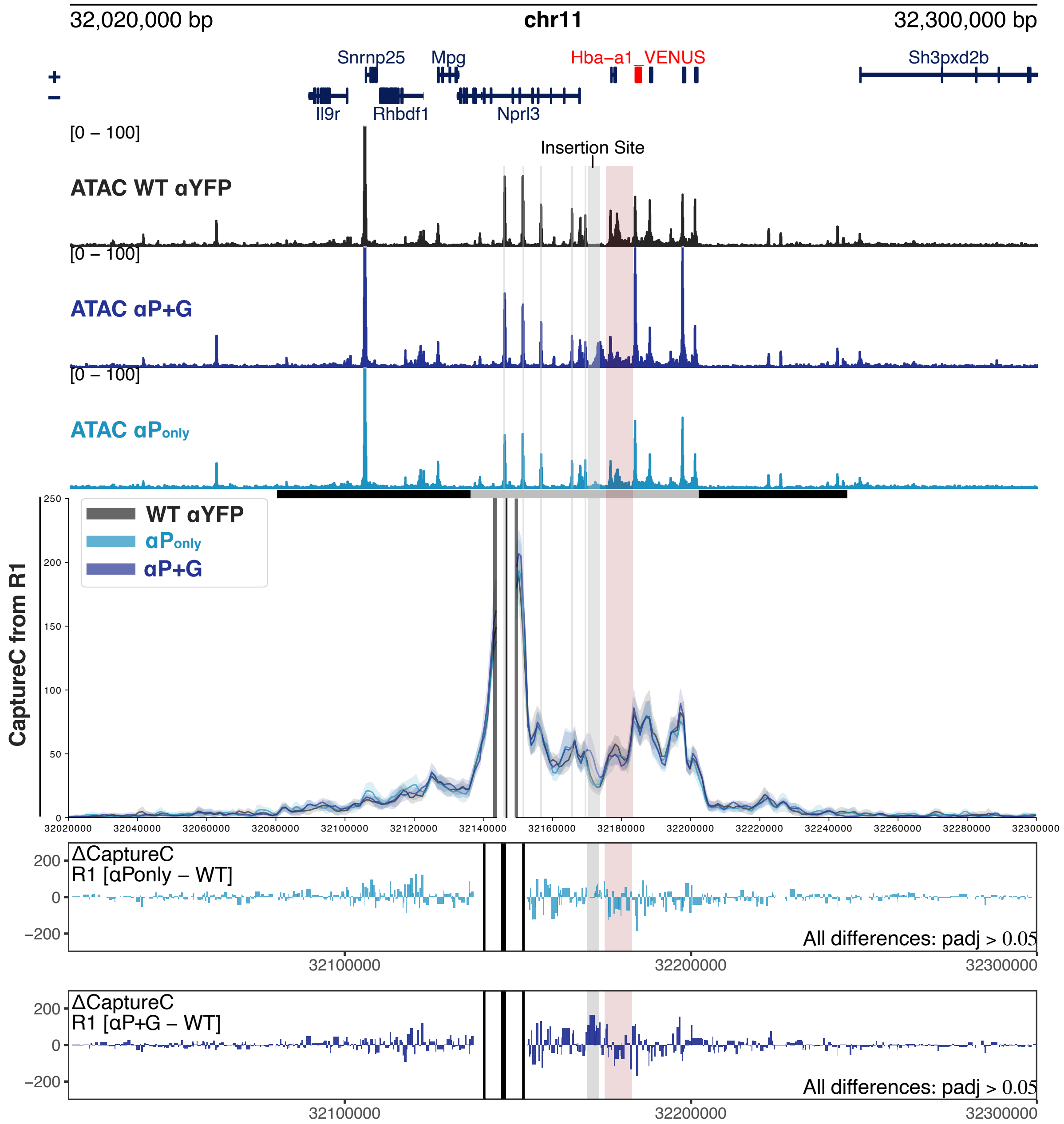

Capture-C from the R1 enhancer in CD71+ erythroid cells derived from edited reporter cells. ATAC-seq in the top panel to show the positions of the elements of interest. Capture-C profiles represent the mean number of normalised unique interactions per restriction fragment from n=3 (WT αYFP), n=4 (αPOnly and αP+G) independent differentiations. Differential tracks (ΔCaptureC) show subtractions (αPOnly - WT αYFP and αP+G-WT αYFP) of the mean number of unique interactions per restriction fragment, scaled to a total of 100,000 interactions in cis. Note differences represented are not determined to be significant (threshold padj>0.05). The region around the insert (mm9: Insert region: chr11:32169922-32173392) is highlighted in grey, and region covering native targets (*Hba-x* - upstream of *Hba-a1* chr11:32175105-32182970) is highlighted in red. To note, read coverage over the *Hba-a1/2* genes is a combination of both inserted and native sources.

Appendix Supplementary information: Limitations on interpretation of Capture-C from insertion models

Whilst DpnII generated Capture-C profiles do not show a significant change in interactions between the inserts and the native genes, there are limitations to this approach used for this context; first, due to the homology of the native genes and inserts, it was not possible to count the most informative reporter, ie the restriction fragment over the α-globin promoter, as reporter reads from the inserted sequences and the native genes are identical. Secondly, a SNP that could have been used to distinguish between native or inserted α-globin promoters was unfortunately positioned too far from the nearest DpnII cut site, hence they were indetectable with this method. Thirdly, due to the sequence similarity of the inserts and native genes, interaction profiles over the native regions may be masked by multimapping of reporters from native and inserted sequences. Therefore, distributions of specific interactions between the enhancers with each promoter remain unclear. This could be rectified with the use of Mnase derived 3C (MCC), in which interaction reporters would not be limited by the position of restriction sites. Despite these limitations, quantification of the regions flanking the informative sites, shows a subtle trend toward an increase of interactions at the insert site in the inserted αP+G model and reciprocal decrease in interactions near the native α-globin promoter which supports the increased accessibility at the insert sites and decrease in *Hba-a1::Venus* expression. Further to this, R2 interactions with *Hba-x* show a subtle decrease in interactions however this is not observed in R1, further highlighting the limitation of this assay when looking for changes of interactions within regions of a few kb.

Appendix Table S1: DNA sequences for sgRNAs, ssODNs and screening primers used for targeting CTCF binding sites at the mouse a-globin locus

Target	Guide Sequence (5’-3’)	ssODN Sequence (5'-3')
θ1	TGGAACGATGCAGCGCCCCC	CGCTCTGCCCGCTGGCTGAGCTCAAAGACGTCCTGAAACACAAGAGGCGGATCCCGCTGCATCGTTCCAGGATGCCTAGGTGTTCACAGATTCTGGTTCAGCTTTGAGCCCTCTGTTTCCCTGGGCTCCCCCTCC
θ2	TGAAACACAAGAGGCCGCCA	CTTGCGCTCTGCCCCCTGGCTGAGCTCAAAGATGTCCTGAAACACAAGAGGCGGATCCCGCTGCATCGTTCCAGGATGCCTAGGTGTTCACAGATTCTGGTTCAGCTTTGAGCCCTCTGTTTCCCTGGGCTCCCT
θ2	GACATCTTTGAGCTCAGCCA	
HS+44	GAAAGCCAGTGGCGCCACCT	N/A
HS+44	CCCTGCAGGCCACTATAAGT	N/A
HS+48	TCCAAGGTCCTCAAGCAGAC	N/A
HS+48	CGACGAGCACCCCCGTGTGG	N/A

Target	Screening primer Sequence (5’-3’)	Notes
θ1	CCCTGGCGGCCTCTTG	
	GTCCAGGACAACAATGCAGC	
θ1	TGTGGAAGACAGAAGAGGGC	
	CTGAAACACAAGAGGCGCTG	
θ2	TTCCGAAGGACTCGGGAAGC	Product is BamHI digested : 206bp & 778bp (Δθ2 & Δθ1θ2 respectively)
	ATGCACAGAGGCAATGCAGC	
HS+44 /+48	TGTCTGGAACTATCTGGAAACTGA	
	GGGAAGAATAGACTAGACACATCA	
HS+48	TGCTTGGCCTTCAGGTTTCA	
	AAGTTCCGAGTCCCGCCAC	

Appendix Table S2: DNA sequences for sgRNAs and regions used for targeting the insertion site.

**Target**	**Sequence (5’-3’)**	**Coordinates in mm9**
5’ sgRNA for insertion site	GCTGTAGTGTAACTAACTGC	
3’ sgRNA for insertion site	GCTTCAAGAACTGCCTTCCTG	
5’ Homology arm		chr11:32170381-32171787
3’ Homology arm		chr11:32171856-32173092
αPromoter	AGGGACCCAGAGGGAGAGGTGGGG<br>GGATGGGCGCTGCTCAGTTTGGTTT<br>GAGGGACTTGCTTCTCTGACCAAGG<br>TAGGAGGATACTAACTTCTTCCCAA<br>ACTGCCATCACTGGAGACGTAGTAA<br>GGGGTAAGAAGTGTGTCCGGGCAA<br>CTGATAAGGATTCCCTGCACCCAGG<br>GGAAGCACAACCCAGCCCCAGAAT<br>CTCAGGGGCCCTAACAAGTTTTACT<br>GGGTAGAGCAAGCACAAACCAGCC<br>AATGAGAACTGCTCCAAGGGCGTGT<br>CCACCCTGCCTGGAGGACACGCCCT<br>TGGAGGGCATATAAGTGCTACTTGC<br>TGCAGGACCAAGACACTTCTGATTC<br>TGACAGACTCAGGAAGAAACCATG<br>GTG	chr11:32183340-32183712
αGene Body	CTCTCTGGGGAAGACAAAAGCAAC<br>ATCAAGGCTGCCTGGGGGAAGATTG<br>GTGGCCATGGTGCTGAATATGGAGC<br>TGAAGCCCTGGAAAGGTGAGAACA<br>GGACCTTGATCTGTAAGGATCACAG<br>GATCCAATATGGACCTGGCACTCGC<br>TCAGTGGGCAGCTTCTAACTATGCT<br>TTTCTGTGACCTCAACTTCTCTTCTC<br>TCCTTCTCCCAGGATGTTTGCTAGCT<br>TCCCCACCACCAAGACCTACTTCCC<br>TCACTTTGATGTAAGCCACGGCTCT<br>GCCCAGGTCAAGGGTCACGGCAAG<br>AAGGTCGCCGATGCTCTGGCCAATG<br>CTGCAGGCCACCTCGATGACCTGCC<br>CGGTGCCCTGTCTGCTCTGAGCGAC<br>CTGCATGCCCACAAGCTGCGTGTGG<br>ATCCCGTCAACTTCAAGGTATGCGC<br>TGGGACCTGGCAGGCGGCATCTGGG<br>ACCCCTAGGAAGGGCTTGGGGGTCC<br>TCGTGCCCAAGGCAGGGAACATAGT<br>GGTCCCAGGAAGGGGAGCAGAGGC<br>ATCAGGGTGTCCACTTTGTCTCCGC<br>AGCTCCTGAGCCATTGCCTGCTGGT<br>GACCTTGGCTAGCCACCACCCTGCC<br>GATTTCACCCCCGCGGTGCATGCCT<br>CTCTGGACAAATTCCTTGCCTCTGTG<br>AGCACCGTGCTGACCTCCAAGTACC<br>GTTAAGCTGCCTTCTGCGGGGCTTG<br>CCTTCTGGCCATGCCCTTCTTCTCTC<br>CCTTGCACCTGTACCTCTTGGTCTTT<br>GAATAAAGCCTGAGTAGGAAGAAG<br>CCTGCA	chr11:32183713-32184493
