# Supplementary material for: A functional overlap between actively transcribed genes and chromatin insulator elements
Source: EMBO J. 2026 Mar 16;45(8):2587–613. doi: 10.1038/s44318-026-00730-2 (PMC13083895; doi:10.1038/s44318-026-00730-2)
Supplement: Supplementary file 8 — Expanded View Figures [file 44318_2026_730_MOESM8_ESM.pdf]

Expanded View Figures

Figure EV1. 3D structure and cis-regulatory elements of the α globin locus.

An expanded view of the α-globin locus in primary Ter119+ erythroid cells. Top panel shows Tiled-C interaction heatmap at 2 kb resolution adapted from (Oudelaar et al, 2020), the horizontal grey bars between the tracks represent the ~70 kb α globin sub-TAD (light grey, mm9 chr11:32,136,000–32,202,000) nested within a larger ~165 kb TAD (dark grey, mm9 chr11:32,080,000–32,245,000), these domains are further highlighted as triangles in the Tiled-C panel. The adult α globin genes are highlighted in red. The individual αlpha globin superenhancer elements (R1, R2, R3, Rm & R4) are highlighted in grey. Below show chromatin characterisation of the region in primary definitive erythroid cells; ATAC-seq (black, this study), H3K27ac ChIP-seq (light green) (Kowalczyk et al, 2012), PolII (dark green) Rad21 (blue) and CTCF ChIP-seq (dark blue) (Hanssen et al, 2017). The orientation of CTCF motifs is shown under peaks by red (forward) and blue (reverse) arrows. Bottom panels show ATAC, CTCF ChIP-seq (labelled with CTCF site identifiers) and Tiled-C interactions zoomed in on the TAD region (mm9 chr11:32,050,000–32,250,000).

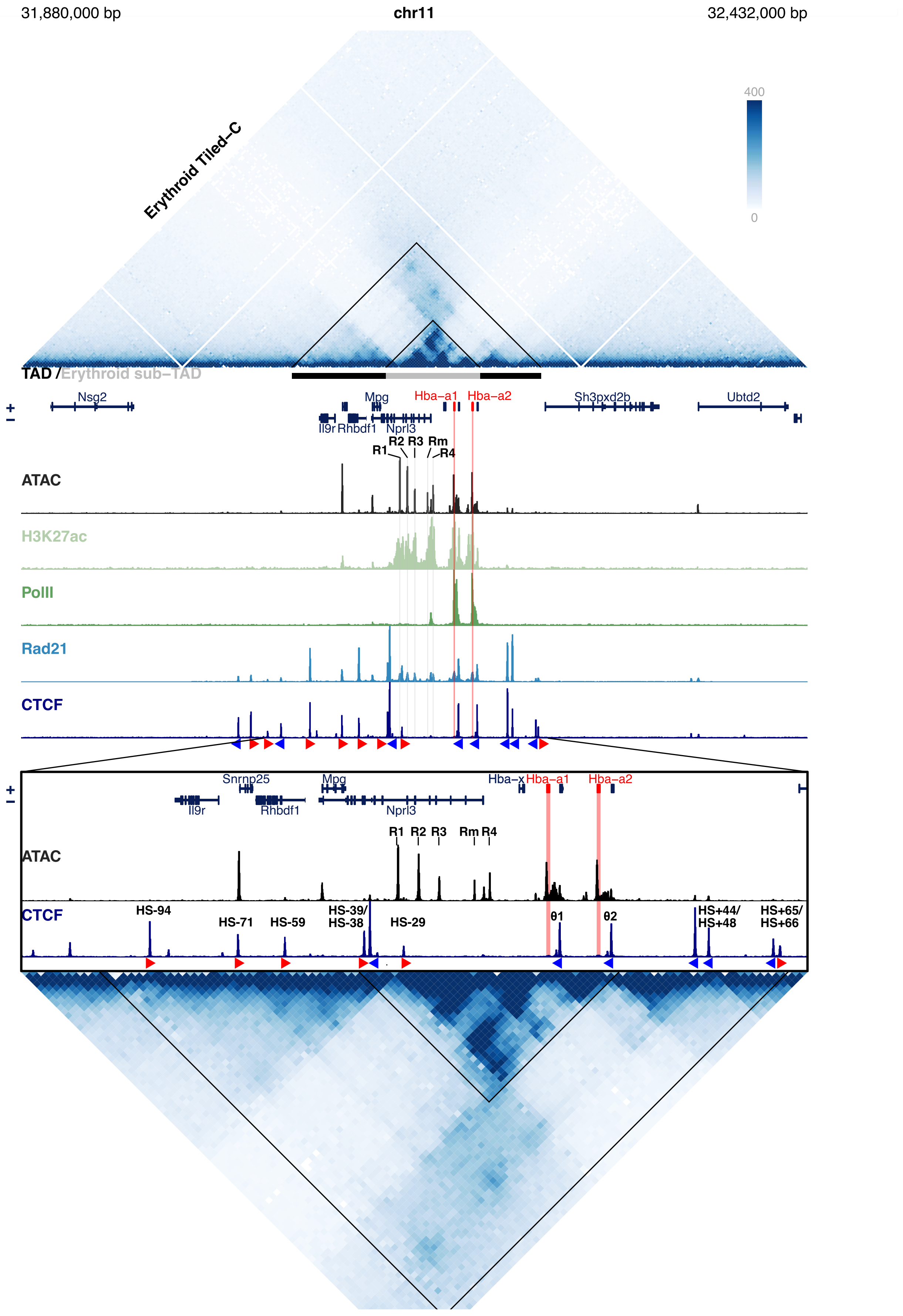

31,880,000 bp
chr11
32,432,000 bp
Erythroid Tiled-C
400
0
TAD / Erythroid sub–TAD
+
–
Nsg2
Mpg
Hba–a1
Hba–a2
Sh3pxd2b
Ubtd2
Il9r
Rhbdf1
Nprl3
R1
R2
R3
Rm
R4
ATAC
H3K27ac
PolII
Rad21
CTCF
Snrnp25
Hba–x
HS-94
HS-71
HS-59
HS-39/
HS-38
HS-29
θ1
θ2
HS+44/
HS+48
HS+65/
HS+66

 © The Author(s)

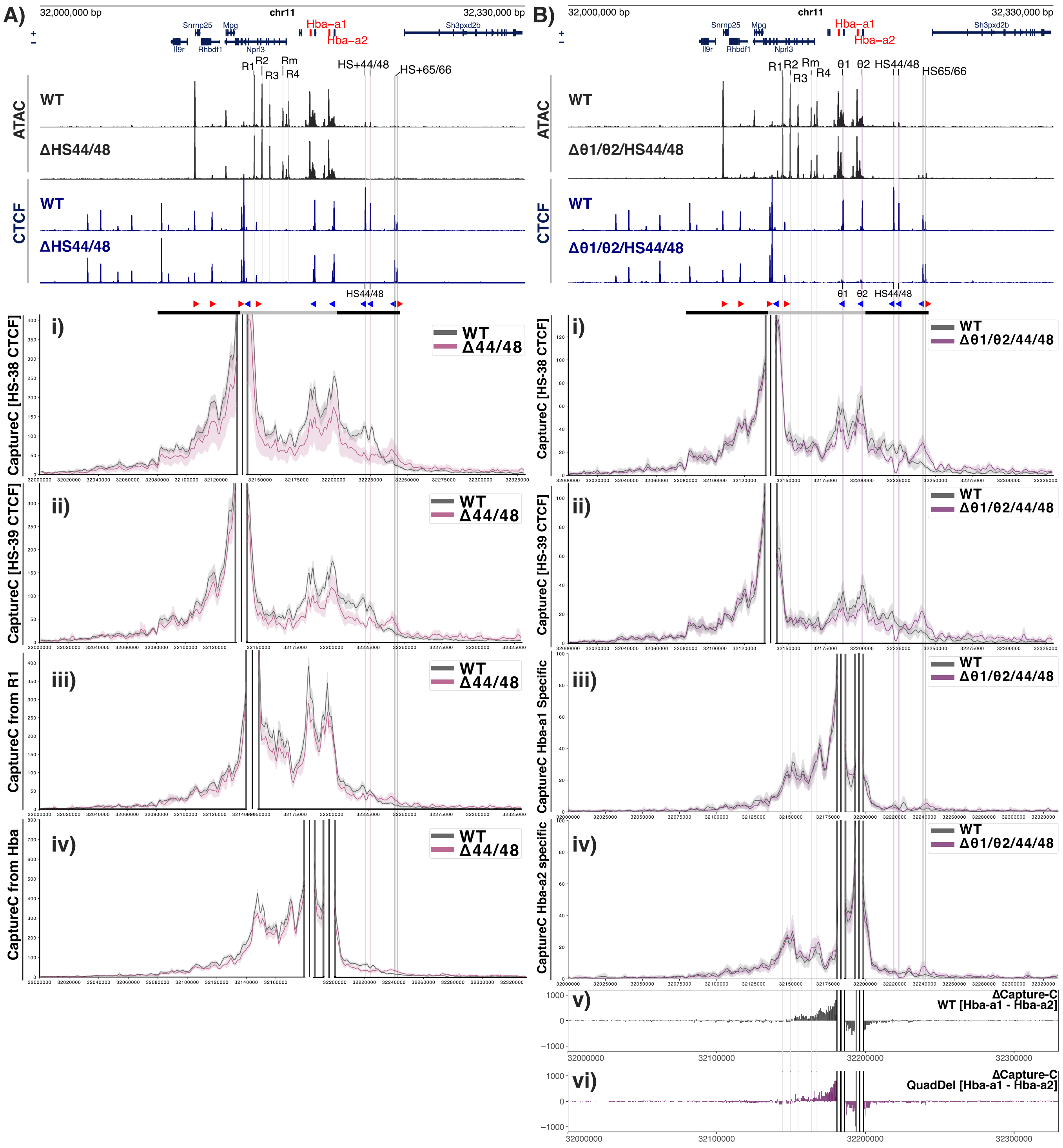
A)
B)
32,000,000 bp
chr11
32,330,000 bp
Hba–a1
Hba–a2
R1 R2 R3 Rm R4
HS+44/48
HS+65/66
θ1 θ2 HS44/48
HS65/66
ATAC
CTCF
WT
ΔHS44/48
Δθ1/θ2/HS44/48
i)
ii)
iii)
iv)
v)
vi)
Δ44/48
Δθ1/θ2/44/48
CaptureC [HS-38 CTCF]
CaptureC [HS-39 CTCF]
CaptureC from R1
CaptureC from Hba
CaptureC Hba-a1 Specific
CaptureC Hba-a2 specific
ΔCapture-C
WT [Hba-a1 - Hba-a2]
QuadDel [Hba-a1 - Hba-a2]

◀ **Figure EV2. Capture-C interaction profiles of the α globin locus from various viewpoints in Δ44/48 and Δθ1/θ2/44/48 erythroid cells.**

(**A**) Top tracks show profiles for ATAC-seq (black) and CTCF ChIP-seq (navy) in primary erythroid cells (Ter119 +) isolated from WT and ΔHS44/48 mice. Profiles show normalised (RPKM) and averaged data from $n = 3$ biological replicates across the α-globin locus (mm9, chr11:32,000,000–32,330,000) with genes and genomic position, with positioning of genes above or below representing sense and antisense transcription, respectively. The adult α globin genes are highlighted in red. The individual α globin superenhancer elements (R1, R2, R3, Rm, and R4) are highlighted in grey. The horizontal grey bars between the tracks represent the ~70 kb α globin sub-TAD (light grey, chr11:32,136,000–32,202,000) nested within a larger ~165 kb TAD (dark grey, chr11:32,080,000–32,245,000). The orientation of CTCF motifs is shown under peaks by red (forward) and blue (reverse) arrows. NG Capture-C interaction profiles of the α-globin locus from WT (grey) and ΔHS44/HS48 (purple) Ter119+ primary erythroid cells, the following viewpoints: (i) HS-38 CTCF, (ii) HS-39 CTCF, (iii) R1 enhancer element and (iv) *Hba-a1/2* genes. The profiles represent normalised and averaged unique interactions from $n = 3$ biological replicates, smoothed with a 1D Gaussian filter. (**B**) As in (**A**) but showing profiles from primary APH-treated spleen cells isolated from WT and Δθ1/θ2/HS + 44/HS + 48 mice. NG Capture-C interaction profiles of the α-globin locus from WT (grey) and Δθ1/θ2/HS + 44/HS + 48 (purple) from the following viewpoints: (i) HS-38 CTCF, (ii) HS-39 CTCF, (iii) *Hba-a1* SNP-specific interactions and (iv) *Hba-a2* SNP-specific. The profiles represent normalised and averaged unique interactions from $n = 3$ biological replicates and halos representing ± standard deviation, smoothed with a 1D Gaussian filter. Differential tracks (ΔCaptureC) show subtractions (v) WT [Hba-a1 – Hba-a2] and (vi) Δθ1/θ2/HS + 44/HS + 48 [Hba-a1 – Hba-a2]) of the mean number of unique interactions per restriction fragment, scaled to a total of 100,000 interactions in cis from SNP-specific counts.

 © The Author(s)

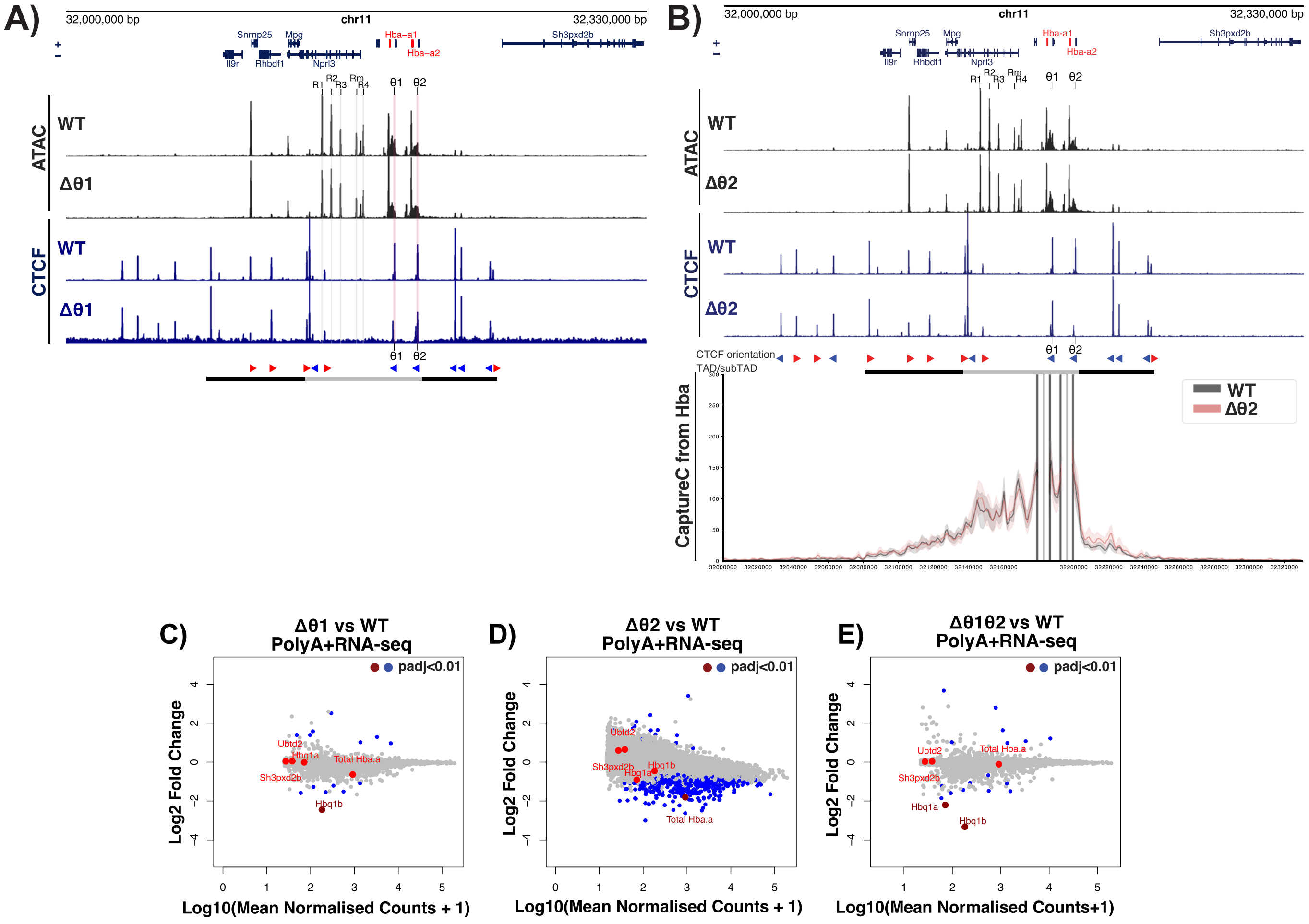

Figure EV3. Chromatin accessibility, CTCF binding and PolyA+RNA-seq in Δθ1 and Δθ2 erythroid cells.

(**A**) Characterisation of Δθ1 primary erythroid cells. Top tracks show profiles for ATAC-seq (black) and CTCF ChIP-seq (navy) in primary erythroid cells (Ter119 +) isolated from WT and Δθ1 mice. Profiles show normalised (RPKM) and averaged data from $n = 3$ biological replicates across the α-globin locus (annotations are as in Fig. 1A). (**B**) Characterisation of Δθ2 primary erythroid cells. Top tracks show profiles for ATAC-seq (black) and CTCF ChIP-seq (navy) in primary erythroid cells (Ter119 +) isolated from WT and Δθ2 mice. Profiles show normalised (RPKM) and averaged data from $n = 3$ biological replicates across the α-globin locus (annotations are as in Fig. 1A). NG Capture-C interaction profiles of the α-globin locus from the combined viewpoint of the *Hba* genes each with an exclusion zone, in WT (grey) and Δθ1/θ2 (pink) Ter119+ primary erythroid cells. The interaction profiles represent normalised and averaged unique interactions from $n = 2$ biological replicates and halos representing ± standard deviation, smoothed with a 1D Gaussian filter. (**C–E**) Differential expression (PolyA + RNA-seq) in Δθ1, Δθ2 and Δθ1/θ2 primary erythroid cells. MAplot of Log2 Fold change versus Log10 of normalised counts in the models above vs WT; each dot represents a gene. Genes with an adjusted *P* value (padj, Benjamini–Hochberg corrected) <0.01 are highlighted in blue. Genes of interest are highlighted in red (3′genes *Sh3pxd2b*, *Ubtd2*, Total Hba (*Hba-a1/2*) and θ1/θ2 associated genes *Hbq1b/a* respectively) and those with a significant difference from WT highlighted with dark red. Results from $n = 3$ biological replicates from each genotype. There is an unexpectedly high number of differential genes in the Δθ2 model further *Hbq1a* appears unchanged upon Δθ2, which is incongruent with the result in Δθ1/θ2. As the Δθ1/θ2 does not show these differences and is a combinatorial deletion of both θ1/θ2 and we must assume these differences in Δθ2 expression are due to technical error.

© The Author(s)

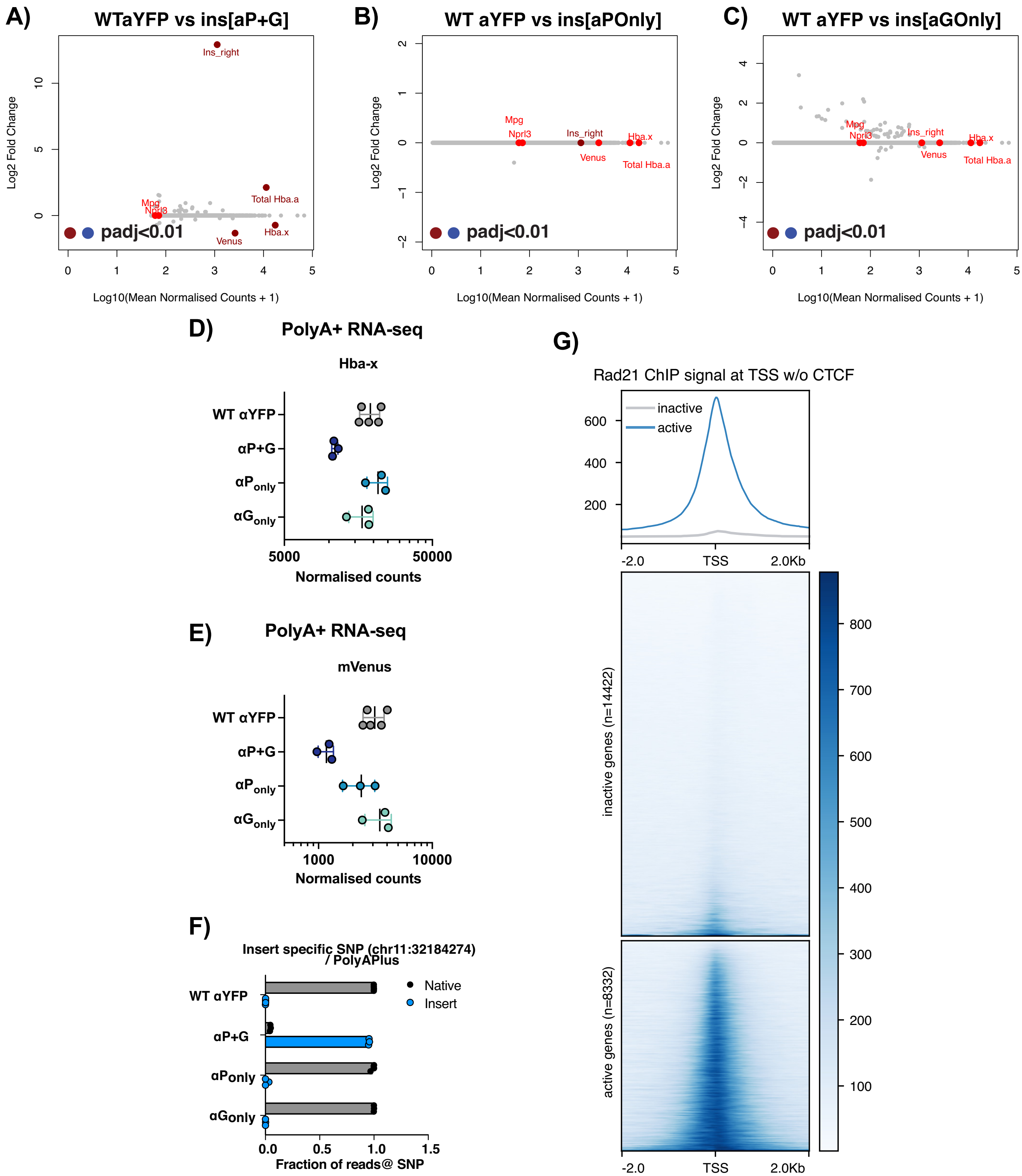
A)
WTaYFP vs ins[aP+G]
Ins_right
Total Hba.a
Mpg
Nprl3
Hba.x
Venus
padj<0.01
Log2 Fold Change
Log10(Mean Normalised Counts + 1)
B)
WT aYFP vs ins[aPOnly]
Mpg
Nprl3
Ins_right
Hba.x
Venus
Total Hba.a
padj<0.01
C)
WT aYFP vs ins[aGOnly]
padj<0.01
D)
PolyA+ RNA-seq
Hba-x
WT αYFP
αP+G
αPonly
αGonly
5000
50000
Normalised counts
E)
PolyA+ RNA-seq
mVenus
1000
10000
Normalised counts
F)
Insert specific SNP (chr11:32184274) / PolyAPlus
Native
Insert
Fraction of reads@ SNP
G)
Rad21 ChIP signal at TSS w/o CTCF
inactive
active
-2.0
TSS
2.0Kb
inactive genes (n=14422)
active genes (n=8332)

Figure EV4. Extended characterisation of in vitro-derived CD71+ cells with inserts.

(**A–C**) Differential expression between the WT aYFP reporter and the insertion models. MAplot of Log2 fold change in gene expression relative to WT against the Log10 of read counts; each dot represents a gene. Highlighted in red are Hba-a (representing total *Hba-a1/2* and aP+G in the P + G model), the region downstream of the insertion site (ins_right) and other genes in the locus. Significant changes plotted with dark red or blue (Wald test *P* value, Benjamini–Hochberg corrected: *P*adj<0.01), non-significant changes are in grey and bright red. Data is $n = 5$ replicates of WT-aYFP and $n = 3$ replicates of each other genotype. As these libraries were not globin-depleted, the representation is skewed toward globin genes. (**D**) *Hba-x* expression as normalised counts in PolyA-Plus RNA-seq. Data is representative of $n = 5$ replicates of WT-aYFP and $n = 3$ replicates of each other genotype. Error bars represent ± standard deviation. (**E**) *mVenus* expression as normalised counts in PolyA-Plus RNA-seq. Data is representative of $n = 5$ replicates of WT-aYFP and $n = 3$ replicates of each other genotype. Error bars represent ± standard deviation. (**F**) Exonic SNP-specific count PolyA-Plus RNA-seq. The aP+G gene had its own unique SNP in exon 3 allowing counting of the proportion of transcripts between the inserted or native α-globin copies. SNPs specific counts of each variant of *Hba* were counted similarly to Fig. 1E in RNA-seq from the CD71+ cells. Error bars represent ± standard deviation. (**G**) Heatmap and summary profile displaying Rad21 ChIP-seq signal in WT in vitro-derived CD71+ erythroid cells at non-redundant transcription start sites (TSS) of inactive (14422) and active (8332) genes, which do not have a CTCF binding site within a 2 kb window around the TSS. Source data are available online for this figure.

© The Author(s)
